# Increasing intensity directly increases the perceived warmth of primary colors

**DOI:** 10.1038/s41598-024-77942-1

**Published:** 2024-11-06

**Authors:** Billy R. Hammond, Colin R. Gardner, Billy R. Wooten, Lisa Renzi-Hammond

**Affiliations:** 1 Vision Sciences Laboratory, University of Georgia, Athens, GA USA; 2 Department of Psychology, Brown University, Providence, RI USA; 3 Institute of Gerontology, College of Public Health, University of Georgia, Athens, GA USA; 4 The University of Georgia, 30602 Athens, GA USA

**Keywords:** Hue-Heat hypothesis, Color, Temperature perception, Psychophysics, Neuroscience, Psychology, Optics and photonics

## Abstract

There is a long history of linking the perceptions of temperature and color (the “Hue-heat hypothesis”): red (R) and yellow (Y) are often considered warm, whereas blue (B) and green (G) are cool. Past studies, however, have largely used relatively broad-band light at a fixed intensity to test these relations. We tested whether increasing the intensity of highly saturated primary colors would lead to a concomitant change in the perceived temperature of those colors. 20 young healthy participants (*M* = 24.80±3.53 years; 45% female; 5% Hispanic; 45% non-White) with normal color vision were tested. An optical system with a Xenon-arc light source, chromatic filters (peak l = 465, 530, 572, 652 nm), and a circular neutral density wedge to vary intensity were used (5 intensity levels). Temperature perception was assessed using an ordinal scale from – 5 (coolest) to + 5 (warmest). The order of the colors used and the intensity levels were varied randomly. Considering the average across intensity levels, B (-1.87) and G (+ 1.09) were considered the coolest, whereas Y (+ 2.1) and R (+ 3.75) were considered the warmest colors. All colors, however, warmed with increasing intensity. A linear regression fit to the averaged data across luminance explained the majority of the variance: B (r^2^ = 0.78), Y (r^2^ = 0.93), G (r^2^ = 0.98), and R (r^2^ = 0.92). Consistent with past data, our results show that color is significantly linked with temperature perception. Increasing the luminance of colors, however, strongly shifts the perception toward increased warmth.

## Introduction

There is a long history of studying how sensory systems interact. What has become clear from such study is that cross-modality is more often the rule than the exception. For example, it is fairly automatic to associate temperature with certain flavors (e.g., cool mint or hot peppers)^1^. This interdependence of perception can be quite meaningful. For example, Wiercioch-Kuzianik et al. (2019)^2^ showed that color was strongly linked to the perception of pain (the nociceptive pathway): painful stimuli that were preceded by the color red were regarded as significantly more painful than when preceded by achromatic stimuli (a blank slide) or other colors. Such connections make sense. From a purely visual perspective, chromatic differences can make objects more visible (e.g., camouflage often takes advantage of color to minimize differences between an object and its surround). Color is also used, however, as a visible signal (e.g., blushing in humans) for behaviors like mating and/or aggression (stimulating preparatory changes in physiology)^3^. Scholkmann et al. (2017)^4^ have shown that short-term exposure to lights of differing color causes linked changes in cerebral hemodynamics/oxygenation and cardiorespiratory dynamics. Exposure to red, for instance, can impair performance in some contexts (e.g., cognitive testing^5^) but enhance it in others (e.g., increasing the probability of winning across a range of competitive sports^6^). 

Of the many effects of color on non-visual responses^7^, one of the more consistent is the association of color appearance with the perception of temperature^8,9^. This is sometimes referred to as the Hue-Heat hypothesis^10^. In its simplest form, it postulates that red and yellow tend to be regarded as warmer colors, whereas blue and green are considered cool. The widest application of this work has been, predictably, in lighting and design^11^. For example, Albers et al. (2015)^12^ varied the color of cabin lighting in aircraft and showed that passengers regarded the cabin as cooler when illuminated with blue dominant as opposed to yellow dominant light (and vice versa, despite keeping the actual temperature constant). A number of studies^13,14^ have shown that varying the correlated color temperature (CCT) of broadband lights causes a predictable change in how individuals perceive ambient temperature (resulting in substantial conservation in the energy required to heat or cool). This kind of work has resulted in the common practice of referring to LEDs with a CCT of about 3000 K (more long-wave light) as “warm white”, whereas a “cool white” has a CCT of about 6000 K (more short-wave light)^15^.

In nearly a century after the first study^16^, however, some predictions of the Hue-Heat hypothesis have not been fully tested. For example, how do participants rate temperature when highly saturated primary colors are used, as opposed to broad-band room illumination? As light intensity increases, how do subjective temperature ratings change? Do blues get colder and reds get warmer? This study was designed to answer these questions. Specifically, we tested the hypothesis that the color primaries blue (B) and green (G) would be rated as cooler than the color primaries yellow (Y) and red (R) and that these perceptions would change as the intensity of the stimuli changed. The four colors we tested (specified in Table 1) were presented in random order and at five randomly presented levels of intensity. Participants rated the perceived temperature of the colors on a scale that ranged from − 5 (cool), 0 (neither warm, nor cool) and + 5 (warm).

**Table 1:** Stimulus characteristics.

Color	Chromaticity coordinates (x, y)	Peak λ	Photometric (cd·sr/m²) levels (1–5)				
Blue	0.14, 0.08	465	26.5	52.8	78.2	108	136
Green	0.25, 0.65	530	24.7	47.2	69.7	96.3	118.5
Yellow	0.39, 0.60	572	34.2	63.7	91.9	124	159.7
Red	0.69, 0.29	652	37.5	73.1	108.1	149.1	175

## Methods

### Participants

A total of 20 participants were recruited from the local Athens GA community (*M* = 24.8±3.53 years; range = 19–32 years; 45% female; 30% Black/African American; 50% White/Caucasian, 15% Asian; 5%White/Hispanic). All participants had uncorrected visual acuity of 20/30 or better in each eye. Participants were excluded based on standard criteria (e.g., past history of ocular disease, color anomalies assessed by Ishiraha plates). All study procedures and materials were approved by the University of Georgia Institutional Review Board prior to initiating the study (PROJECT00008575). All participants gave both written and verbal informed consent prior to participation, the tenets of the Declaration of Helsinki, and the International Conference on Harmonization Good Clinical Practice E6 (ICH-GCP) were adhered to at all times during the execution of the study.

### Stimuli

**Figure 1:**
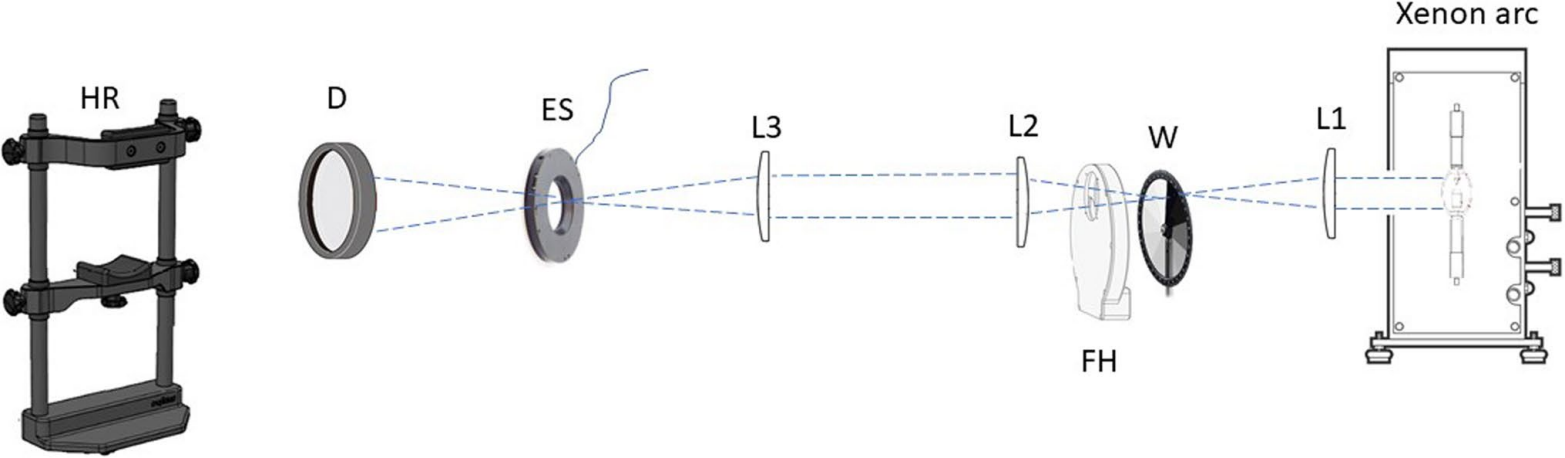
The optical system used for presenting the color stimuli at differing energy levels. The first lens (L1) focuses light through a circular neutral density wedge (W). That light passes through the colored filters placed in a rotating filter holder (FH). Light is relayed by the second (L2) and third lens (L3) through an electromagnetic optical shutter (ES) with an associated timer. The viewing surface for the subject (eye position stabilized with a chin and forehead rest, HR) is an optical diffuser (D).

An optical system (see the schematic in Fig. 1) was used with a 1000-W xenon arc light source (Newport Corp; Irvine, CA; newport.com). Light from this source was alternatively focused and collimated using mounted plano-convex achromatic lenses. Stimuli were rendered chromatic (see details in Table 1) using glass color filters (optical filter glass; www.Schott.com). The intensity of the light was varied using a circular neutral density wedge (Newport Corp; Irvine, CA; newport.com). Shuttering of the light was accomplished using a high-speed electromechanical shutter and timer (Vincent Associates; Rochester, NY; uniblitz.com). Stimuli were viewed on a mounted thin-glass diffuser placed in a collimated portion of the beam 9.3 cm from the plane of the eye. Stray light was minimized by optical baffling. The system was calibrated using a spectral radiometer (ILT 950, International Light Technologies; Peabody, MA; internationallight.com). As shown in Table 1, the most intense setting across colors was 175 cd·sr/m² (irradiance = 37 µW/cm^2^) which, under these conditions (dark room, close to the eye, etc.) was considered by some participants as uncomfortably bright. In our pilot testing, we used this subjective impression to determine our most intense setting and then stepped down in intervals approximating about 0.3 log units.

### Procedure

Participants were shown four colors, B, Y, G and R (see Table 1) presented as a circular test field subtending 13 degrees while their head positions were stabilized with a chin-and-forehead rest assembly. Colors were presented in random order for each participant, with two-second exposures followed by two-second delays. Stimulus exposures were continued for as long as participants needed in order to render their rankings. Intensity levels (see Table 1) were also varied randomly within each color. Participants were asked to rate the temperature of each color using an ordinal scale where − 5 was the coolest ranking; zero indicated neither warm, nor cool; and + 5 indicated the warmest ranking (an achromatic placard was placed on the wall in easy view of the participants as a reminder of the scale). The overall adaptation state of the participants was not controlled.

## Results

**Figure 2:**
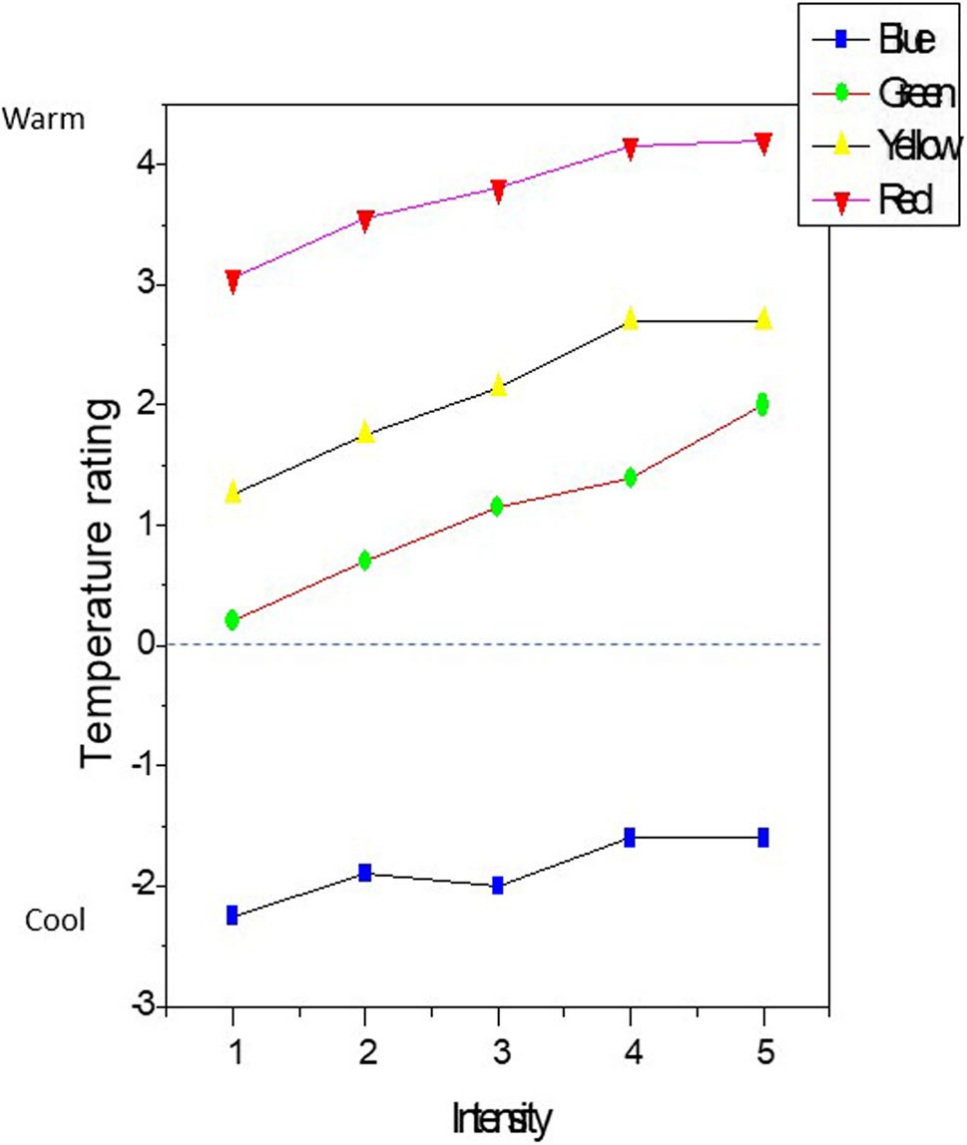
The four color conditions plotted by intensity levels.

On average, the participants rated the perceived temperature of the four colors according to the typical pattern. For example, at the lowest intensity, the average temperature ratings were B = -2.25, G = + 0.2, Y = + 1.25, R = + 3.05. A one-way ANOVA showed that these temperature ratings were significantly different across colors (F_[3, 76]_ = 22.7, *p* < 0.0001). Post-hoc analyses revealed that each color significantly differed from every other color (*p* < 0.0001). This difference was maintained across intensity levels. Blue was always ranked the coolest and red was always ranked the warmest, but participants were less variable in their rankings of warmth as wavelength increased. For example, the average standard deviation across all 5 intensity levels for blue (3.18) was double the average standard deviation across all 5 intensity levels for red (1.04). Yellow was generally ranked closer to red on the warm side (with a similar average standard deviation across all 5 intensity levels; 1.32) and green was ranked closer to neutral with an average standard deviation across all 5 intensity levels of 2.17. These data are shown in Fig. 2.

**Figure 3:**
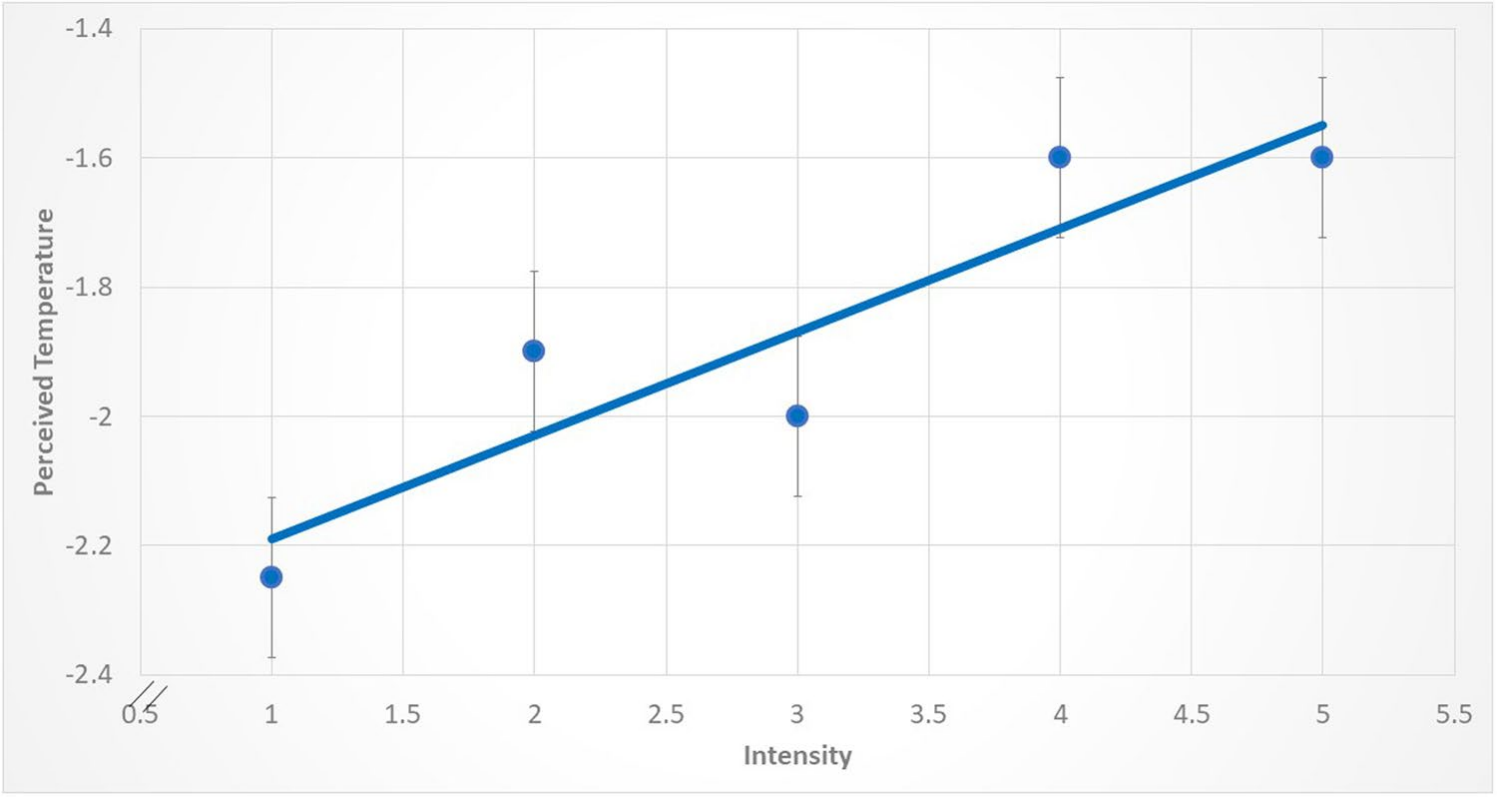
The relation between temperature ratings in the blue condition and changes in the intensity of the stimulus. The points represent the averages and the lines are the standard error of the mean. Each data point represents the average across participants. The data are fit by a linear regression (y = -2.35 + 0.16x, r^2^ = 0.78, *p* < 0.03).

**Figure 4:**
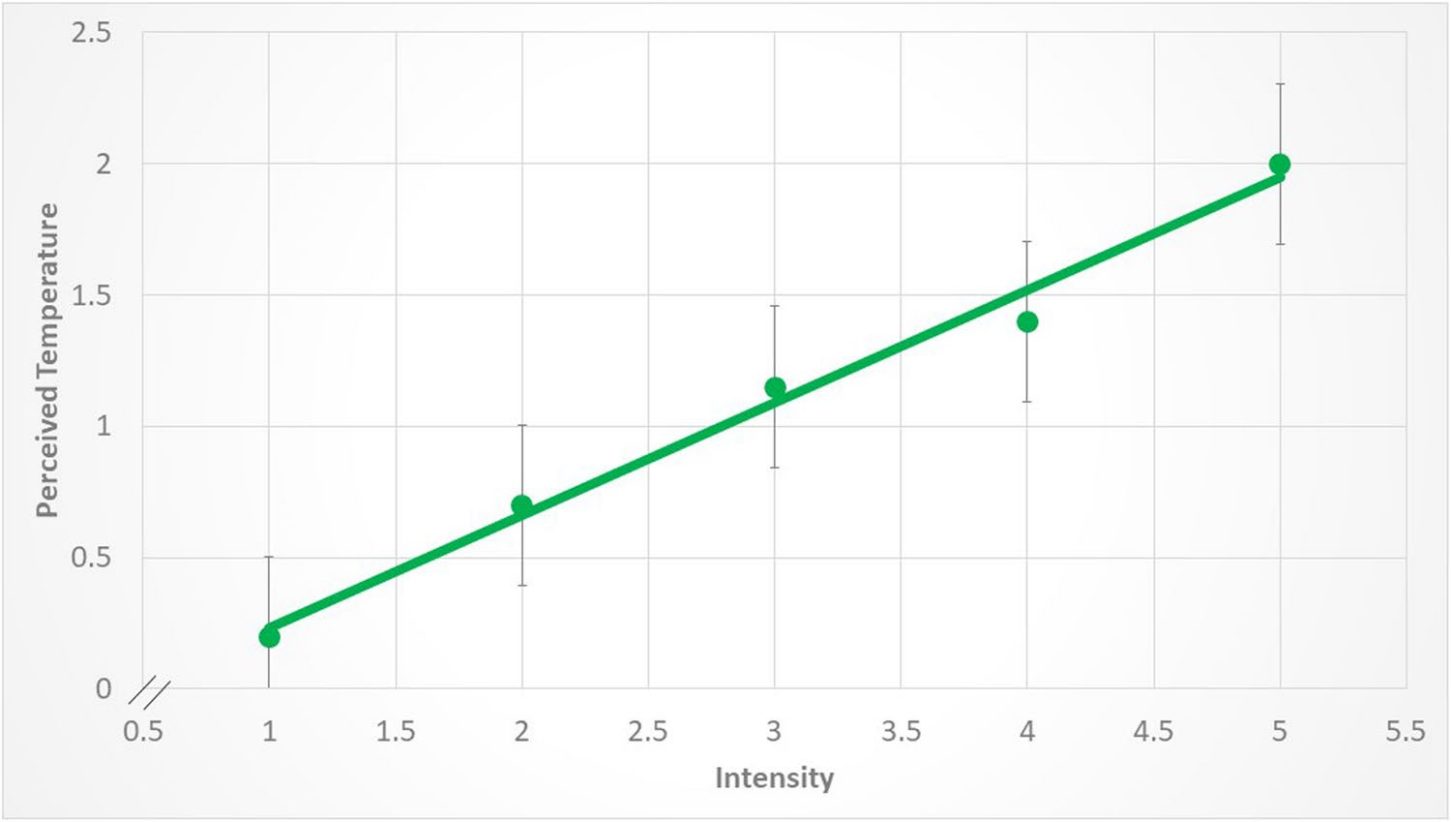
The relation between temperature ratings in the green condition and changes in the intensity of the stimulus. The points represent the averages and the lines are the standard error of the mean. Each data point represents the average across participants. The data are fit by a linear regression (y = -0.2 + 0.43x, r^2^ = 0.98, *p* < 0.0001).

**Figure 5:**
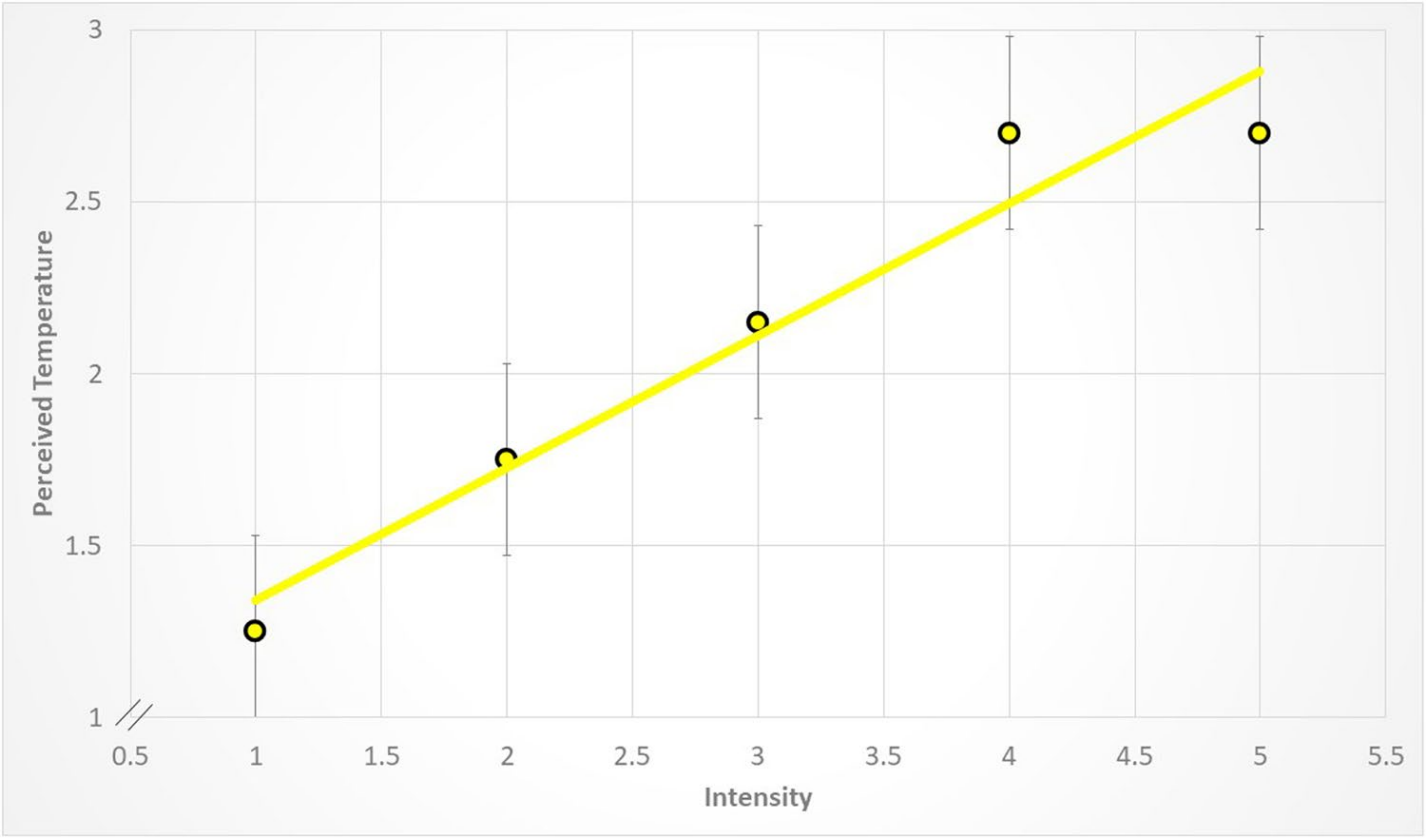
The relation between temperature ratings in the yellow condition and changes in the intensity of the stimulus. The points represent the averages and the lines are the standard error of the mean. Each data point represents the average across participants. The data are fit by a linear regression (y = 0.96 + 0.39x, r^2^ = 0.93, *p* < 0.005).

**Figure 6:**
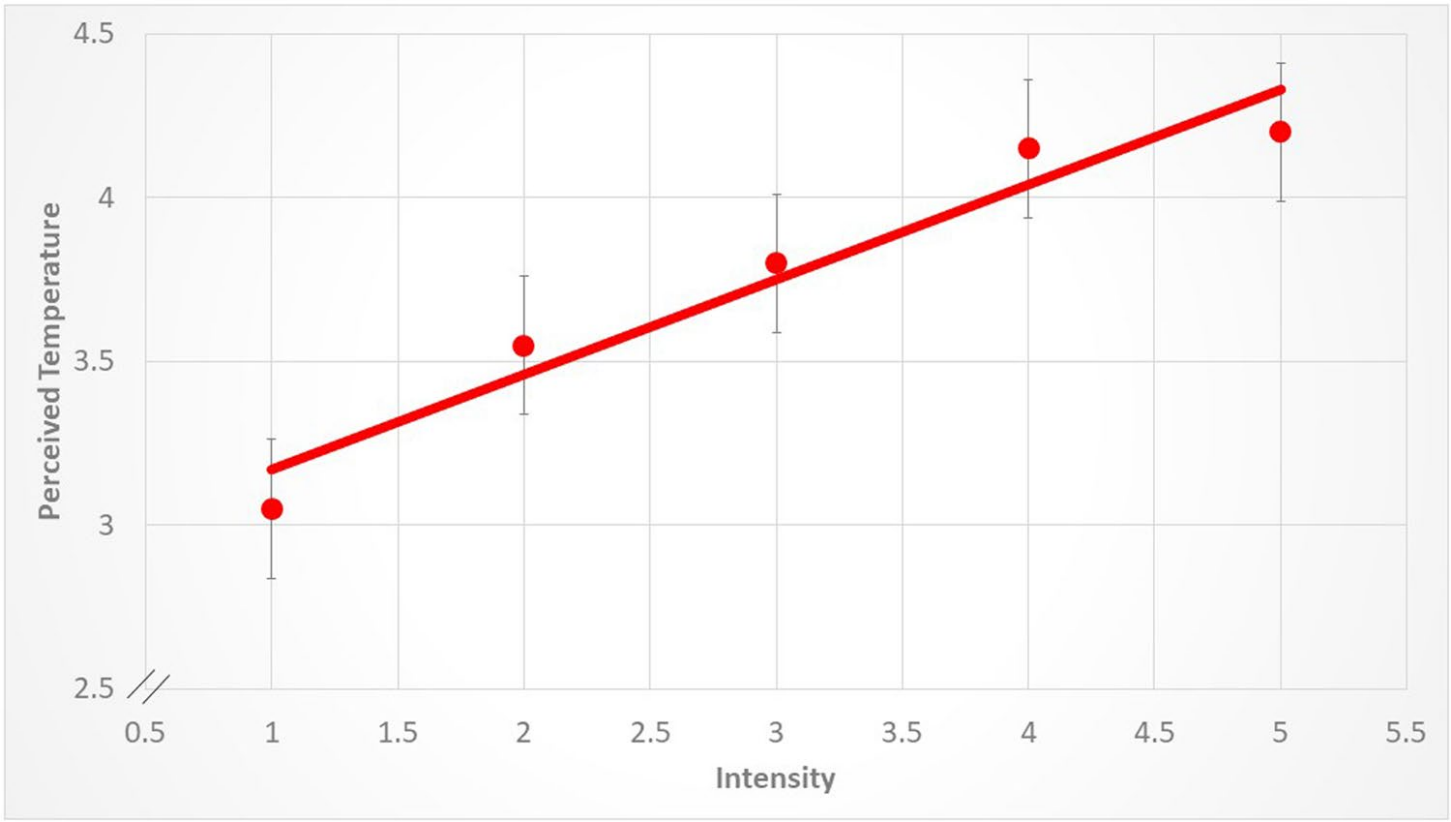
The relation between temperature ratings in the red condition and changes in the intensity of the stimulus. The points represent the averages and the lines are the standard error of the mean. Each data point represents the average across participants. The data are fit by a linear regression (y = 2.88 + 0.29x, r^2^ = 0.92, *p* < 0.006).

Despite, however, these classic rankings (e.g., blue being ranked cool and red being ranked warm), increasing the luminance of the stimulus had a very strong and uniform effect on the perceived temperature, across colors. This is shown in Figs. 3, 4, 5 and 6. For example, increasing the intensity of the coolest color (blue) shifted the rankings from a low of -2.25 to a high of -1.6. Red, the warmest color, shifted from 3.05 to a high of 4.2. These changes in perceived temperature were strongly linear. For example, as shown in Fig. 4, a linear regression explained nearly all of the variance in the averaged data (*r*^2^ = 0.98). As shown in Figs. 3, 4, 5 and 6, the variability between participants was about twice as high for the blue and green stimuli as compared to red and yellow. For example, the average SEM for blue was 0.71, whereas for red it was 0.23.

## Discussion

We found two main effects in this study. The first effect (between-colors effect) was that as wavelength increased, regardless of the intensity tested, colors were perceived as “warmer.” Blue and green stimuli were significantly perceived as cooler, and yellow and red stimuli were perceived as warmer, irrespective of intensity. As shown in Fig. 2, there was little overlap between the four colors that we tested. Likely the most parsimonious explanation for these results, which are consistent with past studies, is that our perception of warmth and coolness is influenced by a lifetime of exposure to colored symbols (colored icons and emojis, hot and cold water taps indicated by red and blue faucets, etc.), and real-world experience As an example of the latter, summer tends to be associated with a bright full (yellow) sun whereas green is often associated with a less hot spring (e.g., the prolific growth of green plants). ).

The second main effect (within-colors effect) was that increasing the intensity of each of the four colors had a uniform effect of skewing perceptions towards warm (see Figs. 3, 4, 5 and 6). For example, blue was always ranked on the cool side, but increasing intensity biased temperature perceptions towards warmth, rather than toward a deeper or more intense perception of coolness. This effect was strong and linear. The amount of variance explained by a linear regression across colors was *r*^2^ = 0.77 (blue), *r*^2^ = 0.93 (yellow), *r*^2^ = 0.98 (green) and *r*^2^ = 0.92 (red).

One explanation for this within-color effect of intensity is that it may also be associative. For example, it is common practice to specify the spectral composition of illuminants with correlated color temperature values expressed in degrees Kelvin. At sufficient intensity, any color can be associated with heat (e.g., a green argon-ion laser). In other words, increasing intensity has a lawful relation with both wavelength and heat (Wien’s displacement law)^17^: when a black body is heated enough, it will glow at wavelengths that include all of the visible spectrum.

There are, however, reasons to suspect that a thermal response to color and intensity is not wholly learned. For example, Katra et al.^8^ showed that for their specific conditions (warm/cool and light/dark ratings of chips from the Natural Color System), the thermal response to color could be precisely predicted by the opponent process mechanisms underlying the color response (i.e., the summed activations of the average B-Y and R-G chromatic valence functions). Exposure to specific colors also has known effects on physiology, which may serve important signaling and/or regulatory functions. One obvious example is the influence of wavelength on endocrine functions utilizing cortisol and melatonin^18^, like circadian rhythms. Blue light, for instance, synchronizes, sleep-wake cycles (peak melanopsin sensitivity is 480 nm)^19^. Exposure to color is thought to influence thermoregulation by the hypothalamus via nonvisual pathways^20^. Exposure to blue light in the evening, for example, alters core body temperature (suppressing pineal gland melatonin activity)^21^. As noted by Kakitsuba et al.^20^. “light intensity would be expected to influence thermal responses such as sweating and shivering.” (page 1).

Using an external signal such as the color of various lights to aide thermal homeostasis makes sense^22^. Thermoregulation is metabolically expensive^23^. The consequences of not effectively matching internal homeostatic mechanisms with surrounding ambient conditions is likely significant. In a recent meta-analysis, Mayes et al. (2023)^24^ showed that most studies confirm that altering the visual environment produced changes in thermal perception. The effect may be bidirectional^25,26^. Kearney (1966)^27^ originally found that ambient temperature also may influence color preferences. In their study participants were exposed to hot (37–43 °C), cool (15–17 °C), or cold (− 5–0 °C) environments with varying light conditions: red (640 nm), yellow (580 nm), green (530 nm), or blue (460 nm). In hot conditions, participants preferred shorter wavelengths like blue/green, whereas in cold conditions, they favored longer wavelengths like red. One distinction with the current study is that participants directly rated their perception of a color’s temperature as opposed to how cool or warm they, themselves felt during the experimental session. Based on past work^28^, one might predict some transfer (e.g., warm colors making an individual actually feel more warm) but this study did not address that question.

In sum, changing both hue and intensity might represent a good strategy for optimizing how an individual may experience temperature: low intensity blue lights seem optimal for inducing the coolest perceptions, whereas high intensity red lights seem optimal for inducing the warmest.

## Data Availability

Individual data collected as part of this study is available by request from the corresponding author.
